# Proximity to the water surface markedly enhances the force production on underwater flapping wings

**DOI:** 10.1371/journal.pone.0299542

**Published:** 2024-03-13

**Authors:** Shantanu S. Bhat, Albert Medina, Fang-Bao Tian, John Young, Joseph C. S. Lai, Sridhar Ravi

**Affiliations:** 1 School of Engineering and Technology, University of New South Wales, Canberra, Australia; 2 U.S. Air Force Research Laboratory, Wright-Patterson Air Force Base, Dayton, OH, United States of America; Dakota State University, UNITED STATES

## Abstract

The potential application of flapping wings in micro-aerial vehicles is gaining interest due to their ability to generate high lift even in confined spaces. Most studies in the past have investigated hovering wings as well as those flapping near solid surfaces. However, the presence of surface tension at the water-air interface and the ability of the water surface to move might differentiate its response to the proximity of wings, compared to that of solid surfaces. Motivated by underwater, amphibian robots and several underwater experimental studies on flapping wings, our study investigated the effects of the proximity of flapping wings to the water surface at low Reynolds numbers (*Re* = 3400). Experiments were performed on a rectangular wing in a water tank with prescribed flapping kinematics and the aerodynamic forces were measured. The effects of surface proximity on the wing in its both upright and inverted orientations were studied. Broadly, the mean lift and drag coefficients in both orientations decreased significantly (by up to 60%) as the distance from the water surface was increased. In the case of the upright orientation, the mean lift coefficient was slightly decreased very close to the water surface with its peak being observed at the normalized clearance of $h/\bar{c}=0.07$. Overall, the study revealed an enhancement in the aerodynamic forces closer to the water surface.

## Introduction

Inspired by insect flight, studies on flapping wings have mostly investigated hovering and forward flights with various wing geometries [1–3] and flapping kinematics [4–6]. Particularly, underwater flapping wings are of great interest to biologically inspired amphibian robots [7, 8] and numerous experimental studies on flapping wings [1, 9–11]. Understanding the effects of a fluid surface on flapping-wing performance is essential for determining control strategies for amphibian robots employing flapping wings underwater. Moreover, the studies on insect-inspired wings utilize underwater experiments of dynamically scaled flapping wings. The use of water as a surrounding medium significantly amplifies the forces acting on the wing compared to those experienced in the air at the same Reynolds number. Thus, more reliable force measurements can be undertaken in underwater experiments, which would have been otherwise difficult to perform in the air due to very low signal-to-noise ratios. However, it is necessary to understand the effects of the proximity of walls and water surfaces in such experiments. In addition, some aquatic species, such as sea butterflies (*Limacina helicina*), maneuver using a flapping-wing-like mechanism [12, 13]. Their Reynolds numbers are similar to those of insects [14] and they approach the surface to eat plankton. Studying the free-surface effects on their underwater flapping wings will provide additional insights into their locomotion and energy expenditure.

A number of previous studies [15–18, for example] have explored the effects of solid surfaces on flapping wings, called the ground and ceiling effects depending on the orientation of the surface with respect to the wings. The initial studies were focussed on 2D flapping wings [19–21, for example] which systematically observed the variations in force coefficients with a change in ground clearance. These experiments were essential since the other simplified models, such as that of Moriche et al. [22], do not account for the ground and ceiling effects. Gao et al. [19] reported for the first time that the trend in the force coefficients with changing ground clearance can be divided into three regimes, namely, the force enhancement, force reduction, and force recovery regimes. For various Reynolds numbers, a 2D wing with pitch amplitude *α*_*m*_ < 45° showed force enhancements at a low chord-normalized clearance of *h*/*c* < 1.5, force reduction in the range 1.5 ≤ *h*/*c* ≤ 4, and force recovery in the regime *h*/*c* > 4 [20]. No recovery regime was observed for high pitch amplitudes [20]. The same three force regimes were also observed in the case of ground effects on a 3D flapping wing [23, 24]. The proximity of the ceiling shows the force enhancement as a result of the increased relative velocity and angle of attack due to the mirroring effect from the ceiling [18, 25].

Compared to a solid surface, a fluid surface can be thought to have different effects as a result of surface tension as well as its ability to move, which still remain under-explored. There are a few studies [26–28] on two-dimensional (2D) foils heaving and pitching underwater. Deng et al. [27] showed that the variation in the mean lift coefficient ${\bar{C}}_{L}$ as a function of the surface clearance is sensitive to the Froude number *Fr*. At a given *Fr*, ${\bar{C}}_{L}$ is observed to increase with the normalized clearance in a lower range $h/\bar{c}<0.7$ and reaches a peak close to $h/\bar{c}∼1.2$, followed by a gradual decrease [28, 29]. At $h/\bar{c}>2$, the free-surface effects are found to be negligible [28]. The increase in ${\bar{C}}_{L}$ at low $h/\bar{c}>2$ has been attributed to wave breaking by [28]. At a high Froude number, *Fr* ≥ 5, Marshall et al. [30] showed a continuous increase in ${\bar{C}}_{L}$ over the range $0\leq h/\bar{c}\leq 3$ with their the potential-flow model for high-speed flow over a submerged flapping plate.

Motivated by the amphibian flapping-wing robots and aquatic locomotion of animals having a similar mechanism, the current study is focussed on the underwater rotational flapping of a three-dimensional (3D) wing. The primary difference between the previous studies discussed above and the current study is that the planar motion of 2D wings does not represent the complex 3D flapping motion about the three Euler angles. All studies on 2D flapping wings include a continuous external unidirectional flow, whereas the surrounding water in the present study is quiescent. The heaving motion in the free-surface-effect studies [26–28] is normal to the free surface, causing the lift force to reverse its orientation in every half cycle. However, a 3D flapping wing generates a vertically upward force in both its half strokes, as described in the insect-inspired studies [4, 31, 32]. The lift generation mechanism in 2D foils is entirely different from that in low-aspect-ratio 3D wings [33]. The aerodynamic force on 3D flapping wings is mostly due to the rotational accelerations that stabilize the leading-edge vortex (LEV) formed over the wing during a half stroke [34, 35] whereas the lift on a foil is largely due to the circulation around the wing and dynamic-stall effects. Furthermore, the cycle-averaged lift on a symmetrically flapping foil is zero whereas the cycle-averaged lift on a symmetrically flapping 3D wing is non-zero as it experiences a positive lift in both the half-strokes. Finally, the potential-flow predictions, such as that of [30], are based on the attached-flow assumptions at low wing angles. Those are unlikely to be observed at high wing angles including flow separation and strong 3D effects. Hence, a separate study on a rotationally flapping 3D wing is necessary to analyze the surface proximity effects on the designs employing such wings.

The present study involves an experimental investigation of the proximity of the water surface on an underwater flapping wing. A rectangular wing was flapped symmetrically underwater at the Reynolds number of *Re* = 3400 with kinematics similar to those of hovering bees and wasps. Precise force measurements were conducted while systematically varying the clearance between the wing’s topmost edge and the water surface. Two arrangements were tested; first, with the wing’s leading edge being on the top, and second, with the inverted wing with its trailing edge being at the top. Overall, the experiments show enhancements in the lift and drag forces acting on the wing when it moves closer to the surface in both arrangements. The experimental method and results are discussed in detail in the following sections.

## Materials and methods

### Flapping-wing setup and wing geometry

**Fig 1:**
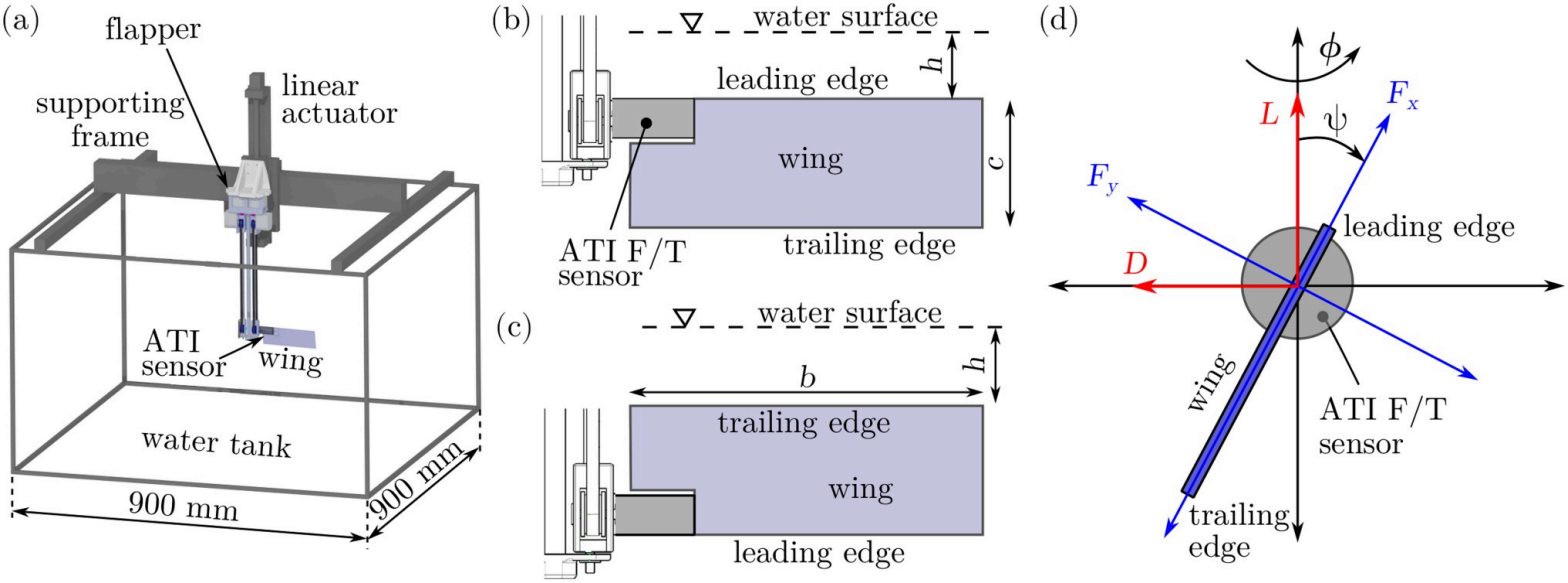
Flapping-wing setup. The schematic of the flapping-wing setup is shown in (a). The orientation arrangement with the wing leading edge being closer to the water surface is shown in (b) and the arrangement with the wing trailing edge being closer to the water surface is shown in (c). The reference coordinate system, angular displacements, and force orientations are shown in (d).

Experiments were conducted on an underwater flapping wing placed in a water tank. The schematic of the setup is shown in Fig 1(a). The flapper was mounted at the center of the tank of size 900 mm × 900 mm × 600 mm using a supporting frame. The flapper has been designed to flap a wing with two degrees of freedom with the help of two identical servo motors (RoboStar SBRS-5314HTG). The first motor controlled the flapping stroke through the main shaft while the second motor controlled the pitch motion through a timing belt and a pulley. The vertical motion of the flapper was controlled by a linear actuator driven by a stepper motor (NEMA 23) with a lead screw of positional accuracy of 0.05 mm.

A rectangular aluminium wing of span *b* = 150 mm and chord *c* = 50 mm was attached to the flapper. A root cutout of 35 mm × 21 mm was applied to accommodate an ATI Nano 17 force and torque sensor at the wing root, as can be seen in Fig 1(b) and 1(c). Consequently, the wing area *S* was reduced and hence, the mean wing chord was calculated as $\bar{c}=S/b$. Due to the pulley and the ATI sensor, the wing root was offset from the rotation axis by *b*_0_ = 20 mm. The details of the wing geometry have been summarized in Table 1(a).

**Table 1:** Wing geometry and kinematic parameters.

(a) Wing geometry			
Span (*b*)	Mean chord ($\bar{c}$)	Area (*S*)	Radius of gyration (*R*_*g*_)
150 mm	45.1 mm	6765 mm^2^	103 mm

(b) Wing kinematics					
Flapping amplitude (*ϕ*_*A*_)	Pitching amplitude (*ψ*_*A*_)	Flapping frequency (*f*)	Flapping profile coefficient (*K*)	Pitching profile coefficient (*C*_*ψ*_)	Re
75°	45°	0.125 Hz	0.8	3.2	3400

Since the experiments were conducted in quiescent water in a tank, there were no flow-obstruction or wake effects. However, the structure at the wing root might influence the wing aerodynamics in two ways: first, it could alter the leading-edge vortex formed during the wing’s sweep and second, it caused the wing to be offset from the rotation axis (by the amount *b*_0_), which affected the wing’s Rossby number (*Ro* = *R*_*g*_/*c*, where *R*_*g*_ is the radius of gyration), ultimately influencing the aerodynamic forces. Bhat et al. examined these two effects in their previous studies [36, 37]. Accordingly, the offset ratios *b*_0_/*b* < 0.25 were found to have minimal effects on the leading-edge vortex. The offset ratio in the current study was 0.13 and thus, was expected to have a minimal effect. Furthermore, a change in the Rossby number can change the aerodynamic forces. The wing root offset will result in lower aerodynamic forces than those without the offset. However, the multi-variable model of Bhat et al. [38] can be used to predict the scaling of the forces for the Rossby-number correction. In the current study, the same wing geometry and offset were maintained across all cases. Hence, the trends in their aerodynamic forces were comparable irrespective of the overall reduction in the forces due to the wing root offset.

### Flapping-wing kinematics

**Fig 2:**
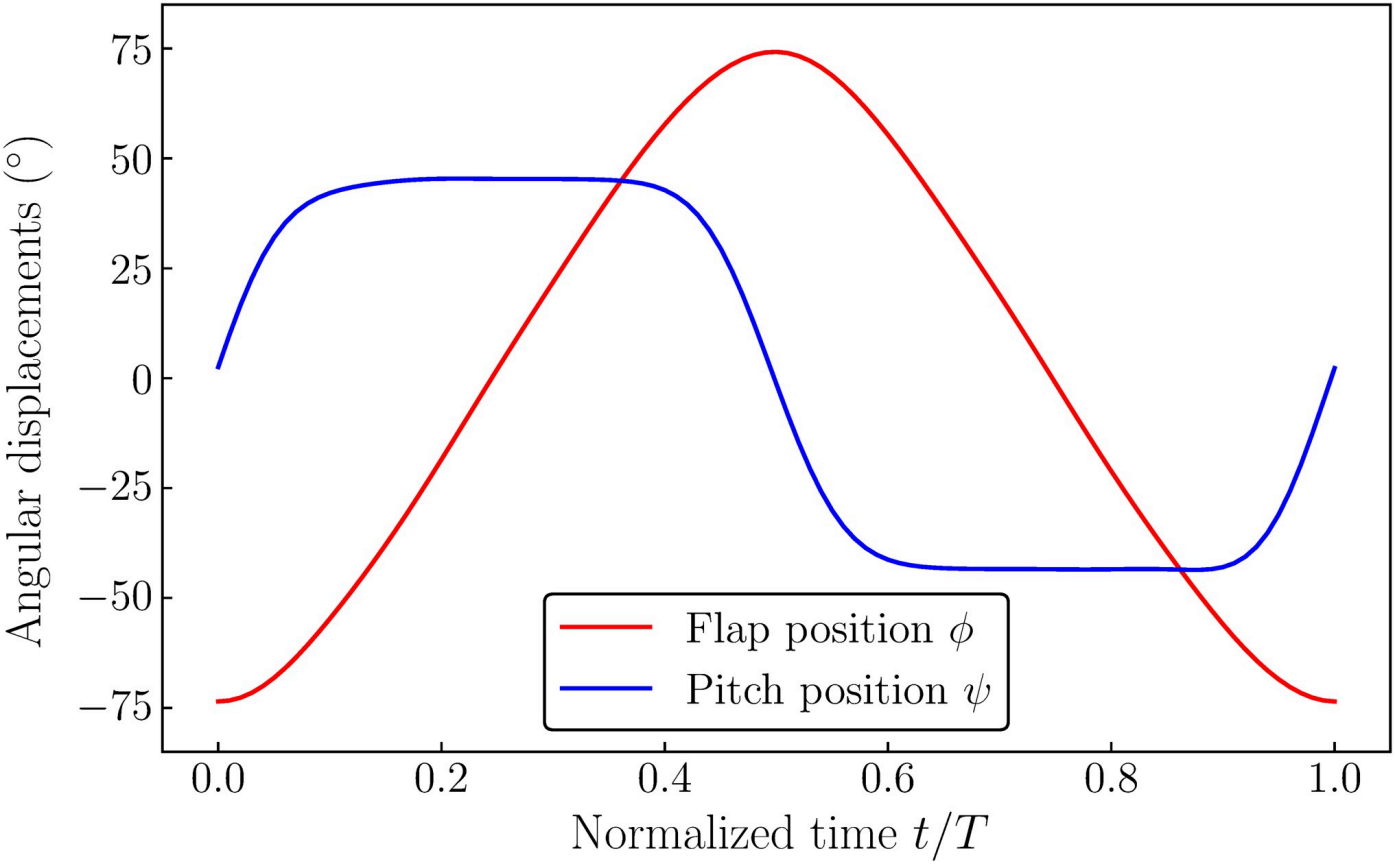
Wing kinematics. The figure shows the wing kinematics prescribed in this study.

Following most studies on flapping wings and insect-inspired wings [33, 39, 40], normal hovering kinematics were applied, in which the wing moved along a horizontal flapping stroke without any deviation from the stroke plane. Here, the wing’s rotation around the vertical axis of the main shaft has been referred to as the flapping stroke and the rotation around the spanwise axis has been referred to as the pitching motion. Note that the pitch angle *ψ* is measured with respect to the vertical axis, as shown in Fig 1(d). The stroke amplitude of *ϕ*_*A*_ = 75°, pitch amplitude *ψ*_*A*_ = 45°, and flapping frequency of *f* = 0.125 Hz were fixed. The resulting Reynolds number was obtained as

$$\begin{array}{c} Re=\frac{\rho \left(4\phi_{A}fR_{g}\right)\bar{c}}{\mu }=3400 \end{array}$$ (1)

where *ρ* and *μ* are the density and viscosity of the surrounding fluid, and *R*_*g*_ is the wing’s radius of gyration. Here, the reference velocity *U*_*g*_ = 4*ϕ*_*A*_*fR*_*g*_ is the mean wing velocity at its radius of gyration. This reference has been known to reduce wing-shape effects [41] and hence, has been used by a number of studies, such as [11, 25, 42–44]. The chosen values of wing aspect ratio $AR=b/\bar{c}$ and *Re* were in the typical ranges observed in bees and insect-sized aerial robots [38, 45, 46]. The flapping stroke and pitch motion profiles were defined, similar to [5, 11], as

$$\begin{array}{c} \phi =\frac{\phi_{A}}{\text{arcsin}K}\text{arcsin}\left[K\text{sin}\left(2\pi ft+\delta_{\psi }\right)\right] \end{array}$$ (2)

and

$$\begin{array}{c} \psi =\frac{\psi_{A}}{\text{tanh}C_{\psi }}\text{tanh}\left[C_{\psi }\text{sin}\left(2\pi ft\right)\right] \end{array}$$ (3)

respectively. Here, *K* is the flapping profile coefficient, *C*_*ψ*_ is the pitching profile coefficient, *t* is the time, and *δ*_*ψ*_ is the pitching phase offset. The values of *K* = 0.8, *C*_*ψ*_ = 3.2, and *δ*_*ψ*_ = *π*/2 were chosen as they were found to result in the best possible power economy in the previous studies [11]. The resulting motion profiles of *ϕ* and *ψ* are shown in Fig 2 and are also summarized in Table 1(b).

### Test procedure

Two arrangements were tested experimentally, with the wing being upright (SLE) and inverted (STE) with respect to the water surface [see Fig 1(b) and 1(c)]. In each arrangement, the clearance between the water surface and the topmost wing edge *h* was initially fixed and the wing was allowed to flap for 10 cycles. The experiment was repeated 3 times at a chosen *h*. The value of the normalized clearance $h/\bar{c}$ was systematically varied between the range [0, 2.2], and the experiments were repeated at every $h/\bar{c}$. The wing was held stationary for 2 minutes between every two experiments to allow the effects of the previously shed vorticity to diminish.

The forces and torques acting on the wing were measured by the ATI Nano17 IP68 F/T sensor at the sampling rate of 1000 Hz using a National Instruments PCI-6143 DAQ board linked to a PC. The ATI sensor was capable of measuring forces in three dimensions with an accuracy of 12.5 mN (i. e. ∼10% of the typical peak-to-peak force magnitudes in this work) and torques in three dimensions with an accuracy of 0.0625 Nmm (i. e. ∼5% of the typical peak-to-peak torque magnitudes in this work). The servo motors’ potentiometer signals were recorded to estimate the wing’s actual angular positions simultaneously with the force data. The forces in the sensor’s frame of reference were resolved to obtain the lift *L* and drag *D*, as shown in Fig 1(d). The recorded raw data were processed using an in-house Python code. The raw data were filtered at the cutoff frequency of *f*_*c*_ = 2 Hz using a fourth-order Butterworth filter.

**Fig 3:**
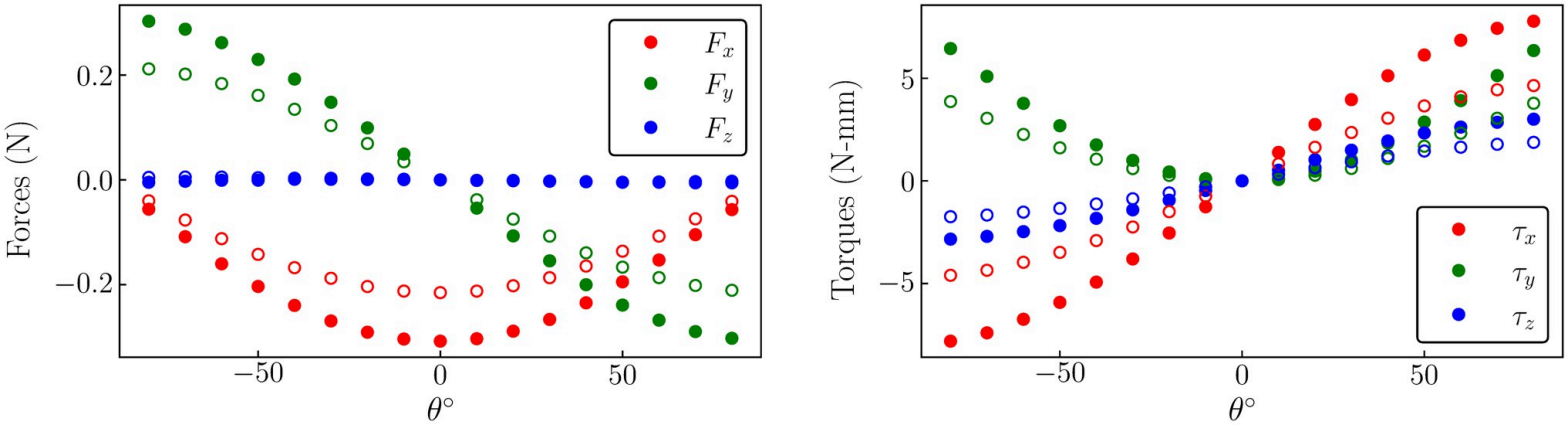
Gravitational loads. The figure shows (a) the forces *F*_*x*_, *F*_*y*_, and *F*_*z*_, and (b) the torques *τ*_*x*_, *τ*_*y*_ and *τ*_*z*_, all in the wing’s frame of reference, at various static wing angles, purely due to gravity. The filled symbols represent the forces and torques measured in air while open symbols represent those measured underwater.

The recorded forces were considered to be the sum of the fluid-mechanical, gravitational, and inertial forces. Thus, to isolate the fluid-mechanical forces, the remaining force components were estimated. To estimate the contribution due to gravitational forces, the wing was held at a constant angle and the forces were recorded in the wing’s frame of reference. This was repeated for various wing angles with both air and water as the surrounding medium. The average forces and torques in the wing’s frame of reference at various static angles are shown in Fig 3. Here, *x* is along the wing chord, *y* is in the wing-normal direction, and *z* is along the wingspan.

**Fig 4:**
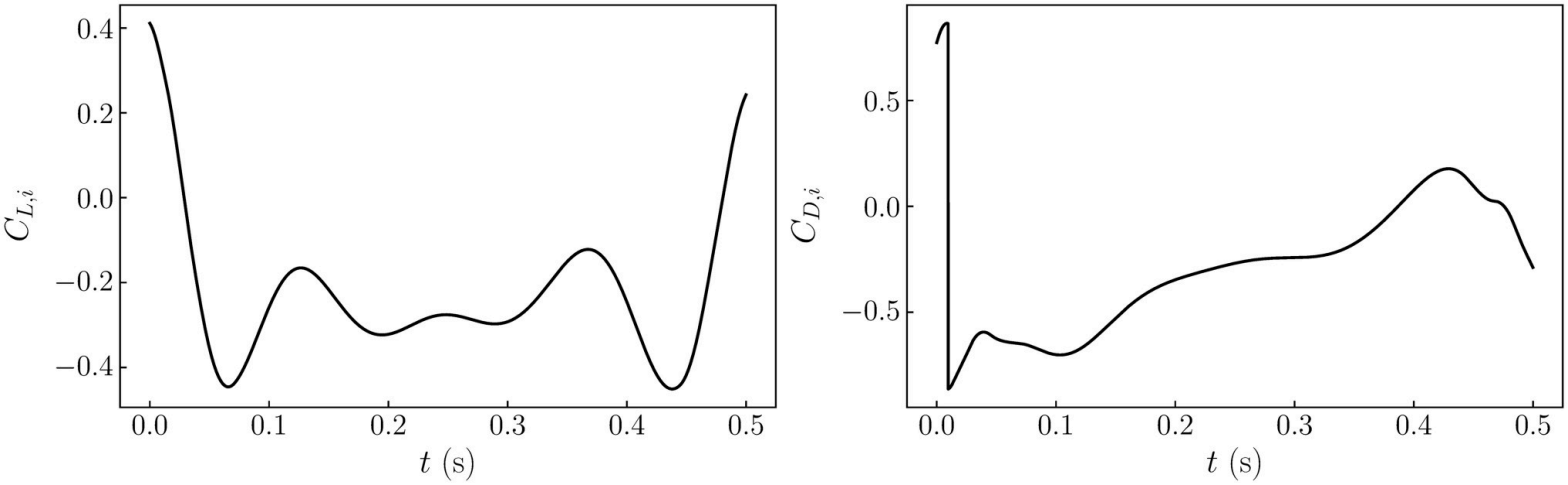
Inertial loads. Time traces of (a) the inertial component of the lift coefficient *C*_*L*,*i*_ and (b) the inertial component of the drag coefficient *C*_*D*,*i*_ are shown over a half stroke.

When the wing was flapped with the prescribed kinematics in air, the fluid-mechanical forces acting on it were negligible compared to the gravitational and inertial forces. Hence, to identify the inertial forces, the gravitational forces, obtained by interpolating the static wing results over the varying wing angle, were subtracted from the measured forces. The time traces of the estimated inertial forces are shown in Fig 4.

Finally, after subtracting the estimated gravitational and inertial loads from the measurements, the data from the 4^th^ to the 9^th^ strokes in all trials at a given $h/\bar{c}$ were phase averaged since the measurements were found to be highly repeatable after 4 strokes (with less than 3% variation). The lift and drag coefficients were computed as $C_{L}=L/\left(0.5\rho U_{g}^{2}S\right)$ and $C_{D}=D/\left(0.5\rho U_{g}^{2}S\right)$, respectively. The results from the instantaneous and mean forces are discussed below.

## Results

### Variations in the instantaneous forces

**Fig 5:**
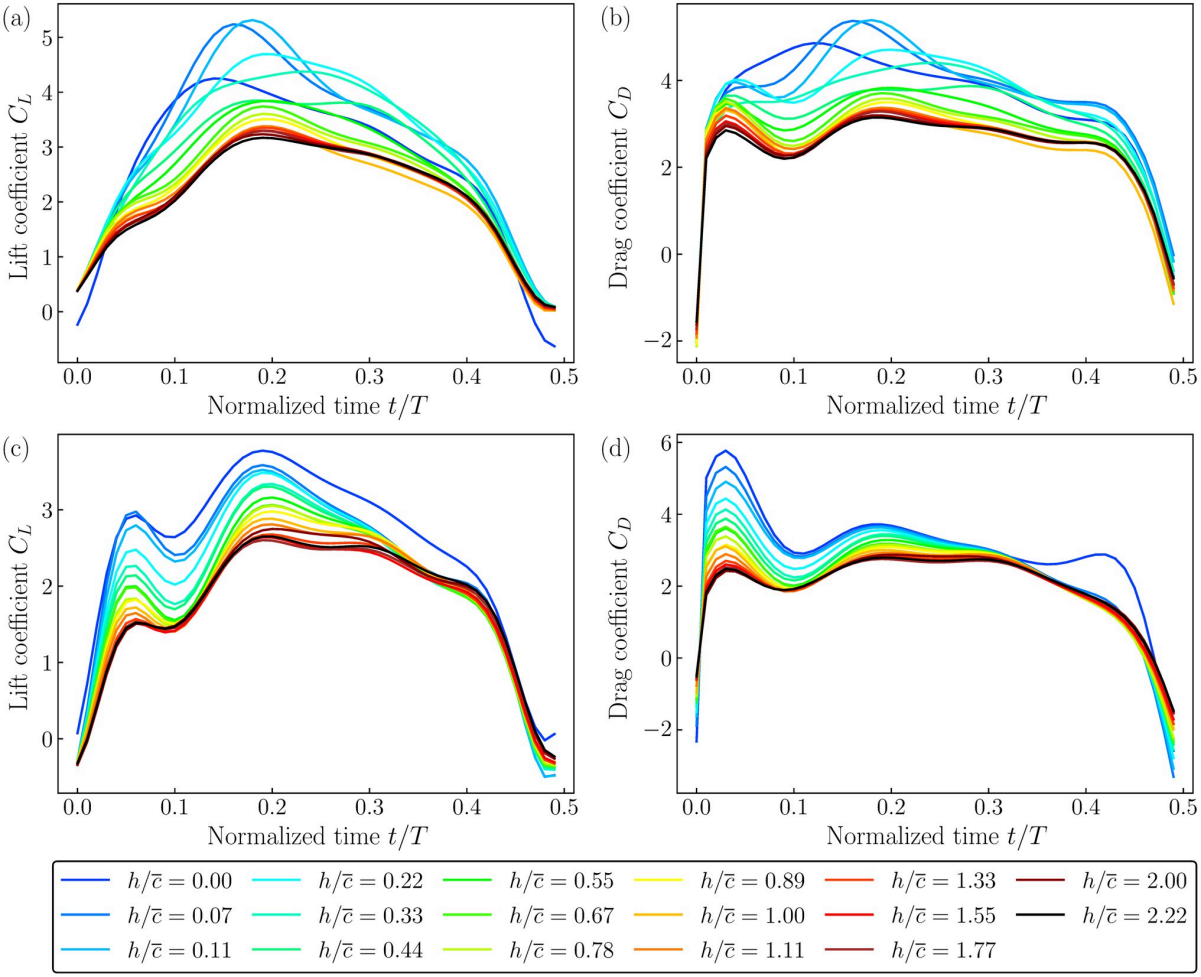
Variations in the force coefficients. For the arrangement with the leading edge pointing towards the surface (SLE), the time traces of *C*_*L*_ and *C*_*D*_ in a half stroke at various $h/\bar{c}$ are shown in (a) and (b), respectively. For the arrangement with the trailing edge pointing towards the surface (STE), the time traces of *C*_*L*_ and *C*_*D*_ at various $h/\bar{c}$ are shown in (c) and (d), respectively.

The time traces of *C*_*L*_ and *C*_*D*_ were obtained from both SLE and STE arrangements at various $h/\bar{c}$, as shown in Fig 5. The corresponding datasets are available in the supplementary dataset (S1 Dataset). In Fig 5, the time traces during a half stroke are presented. The variations in *C*_*L*_ and *C*_*D*_ are found to be similar to those obtained by [11, 47] with similar wing kinematics. *C*_*L*_ and *C*_*D*_ values reach the maximum close to the midstroke, where the instantaneous pitch angle is *ψ* = 45° and the flapping stroke velocity approaches the maximum. At this *t*/*T*, the leading-edge vortex on the wing is expected to be fully developed and stabilized, which would be responsible for creating the maximum suction pressure beneath itself, resulting in high drag and lift values. Beyond *t*/*T* = 0.25, the wing decelerates and hence, both *C*_*L*_ and *C*_*D*_ also start decreasing, as expected. Note that the peak values of *C*_*L*_ and *C*_*D*_ were observed to be reached before *t*/*T* = 0.25.

The time variations in *C*_*L*_ and *C*_*D*_ can be explained by the contributions of the translational and rotational effects, as described by the quasi-steady models on flapping wings [48–50]. According to these models, the translational effects are prevalent during the wing’s sweep motion while the rotational effects are prevalent at a high pitch-angular velocity. The contribution of the translational effects to *C*_*L*_ is proportional to $\dot{\phi }$ as well as sin(2*α*) [49]. Thus, *C*_*L*_ is expected to reach its peak at the midstroke when $\dot{\phi }$ is maximum and *α* = 45°, as has been observed in the current case. Although the variation in $\dot{\phi }$ is sinusoidal, the variations in *C*_*L*_ and *C*_*D*_ are non-sinusoidal due to the additional effects from the rotational forces which are proportional to $\dot{\alpha }$ for any given $h/\bar{c}$.

In both SLE and STE arrangements, as the wing moves away from the surface, indicated by an increase in $h/\bar{c}$, the values of *C*_*L*_ and *C*_*D*_ start to decrease. At higher $h/\bar{c}$, they appear to approach the time traces obtained at $h/\bar{c}=2.22$ with only minimal changes. However, the overall change in the peak values is approximately 50%, which is significant. It might be hypothesized that the occurrence of increased *C*_*L*_ and *C*_*D*_ near the surface might be due to the stronger LEV formed between the limited gap of fluid available over the wing. The accelerated flow in the smaller clearance might be responsible for creating higher shear and a stronger LEV, similar to that observed in the ground and ceiling effects [18]. Nevertheless, this data clearly shows a significant influence of the proximity of the wing to the water surface on the aerodynamic forces.

### Effects on the mean performance

**Fig 6:**
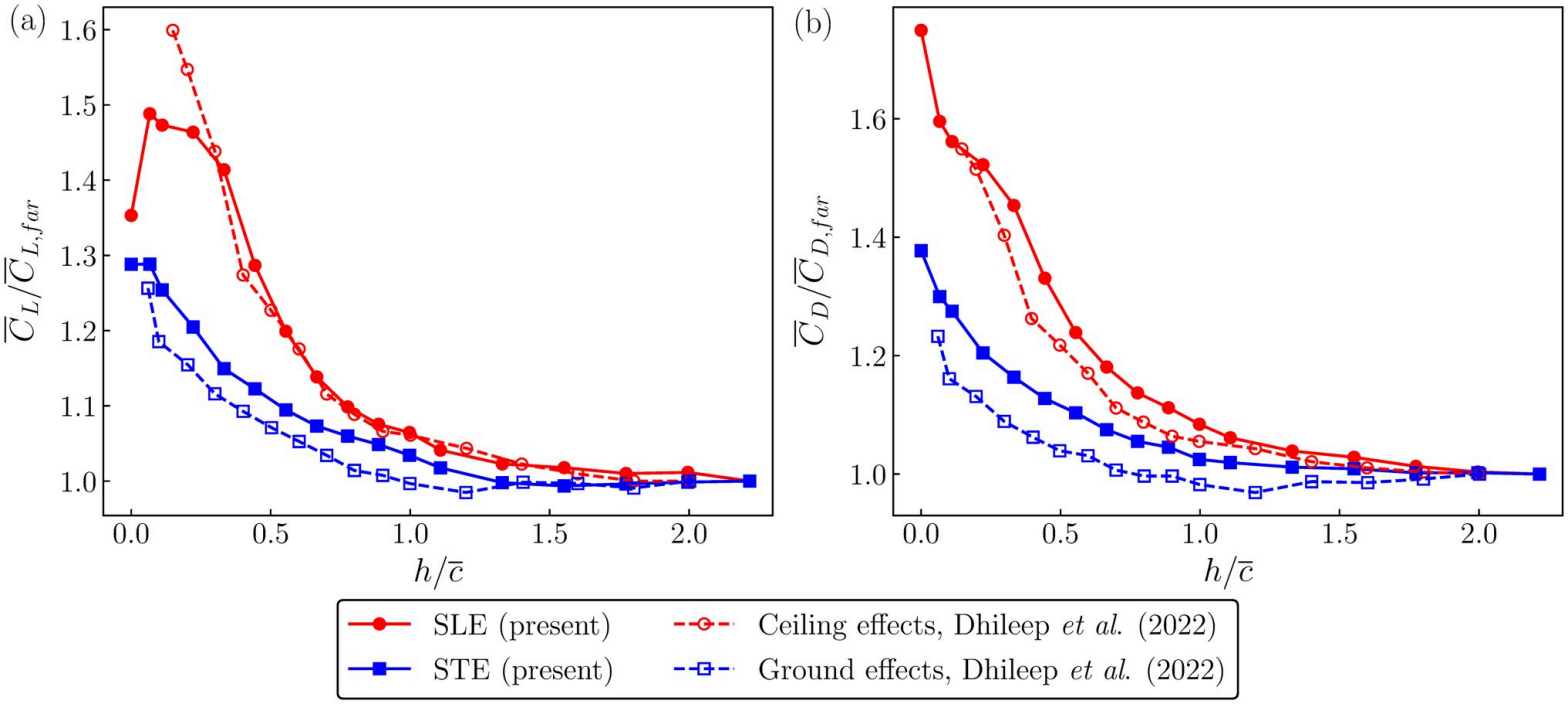
Variations in the mean force coefficients. The variations in (a) the mean lift coefficient normalized by the far-field mean lift coefficient (${\bar{C}}_{L}/{\bar{C}}_{L,far}$) and (b) the mean drag coefficient normalized by the far-field mean drag coefficient (${\bar{C}}_{D}/{\bar{C}}_{D,far}$) with the normalized height ($h/\bar{c}$) are shown. The present data with SLE and STE arrangements are compared with the data for the ground and ceiling effects reported by [18].

The trends in the wing performance with varying proximity to the surface can be better analyzed by observing the cycle-averaged lift and drag coefficients, i.e. ${\bar{C}}_{L}$ and ${\bar{C}}_{D}$, respectively. For a better comparison, the values of ${\bar{C}}_{L}$ and ${\bar{C}}_{D}$ at various $h/\bar{c}$ have been normalized by the corresponding far-field values at $h/\bar{c}=2.2$, i.e. by ${\bar{C}}_{L,far}$ and ${\bar{C}}_{D,far}$, respectively. Fig 6(a) and 6(b) shows variations in ${\bar{C}}_{L}/{\bar{C}}_{L,far}$ and ${\bar{C}}_{D}/{\bar{C}}_{D,far}$, respectively, against $h/\bar{c}$ for both SLE and STE arrangements. Potentially, replacing the water surface with a rigid surface would make the SLE experience ceiling effects and the STE experience ground effects. Hence, the data for ceiling and ground effects for a wing flapping at a similar *Re* (*Re* = 3600) from [18] are also compared.

The SLE arrangement can be compared to the ceiling effects studies with the difference that the surface in the SLE case is deformable. The ceiling-effects studies on 3D flapping wings [18, 25, 51] report a monotonic increase in ${\bar{C}}_{L}$ with a decrease in $h/\bar{c}$. Meng [25] has attributed this to the increased relative velocity and effective angle of attack due to the interaction between the LEV and the opposite-sign vorticity as a result of the mirroring effect from the surface. The increased suction pressure beneath the LEV at a given velocity and angle is the primary contributor to the aerodynamic forces [34]. The same reason would have caused ${\bar{C}}_{L}$ to increase with $h/\bar{c}$ in the current case. However, at sufficiently low $h/\bar{c}$, the free surface would be highly deformed, and thus, the mirroring effect would be deflected at an angle, making the relative velocity and effective angle to gradually reduce. Thus, the peak ${\bar{C}}_{L}$ is observed with a clearance of $h/\bar{c}=0.07$. In the STE case, however, a monotonic decrease in ${\bar{C}}_{L}$ is observed. This might be because, in this case, the leading edge is further away from the surface, having reduced influence from the mirroring effects than in the SLE case. In both SLE and STE cases, the values of ${\bar{C}}_{L}$ and ${\bar{C}}_{D}$ approach those for the far-field case with minimal surface effects.

Overall, our measurements show a significant influence of the proximity of the water surface on the lift and drag coefficients. The variation in the mean aerodynamic forces with the clearance shows a different trend very close to the free surface compared to that observed with a solid surface or ceiling. Thus, these results might be of great interest to the designers of the amphibian and underwater flapping-wing robots. The sudden force enhancements after approaching the water surface will require appropriate controls to be applied to those robots. Moreover, the present study will also provide guidelines to maintain an appropriate depth underwater for conducting reliable experiments on flapping wings.

## Conclusion

The aerodynamic performance of a flapping wing in close proximity to the water surface was investigated experimentally at *Re* = 3400. The wing was prescribed to undergo symmetric hovering kinematics with the stroke amplitude of 75° and pitch amplitude of 45°. The performance was analyzed in terms of the lift and drag coefficients measured during the experiments. The wing was tested with upright (the leading edge pointed towards the surface, i.e. SLE) and inverted (the trailing edge pointed towards the surface, i.e. STE) orientations and the clearance between its topmost edge and the water surface (*h*) was systematically varied.

The time traces of the lift and drag coefficients were observed to undergo similar trends irrespective of the normalized clearance $h/\bar{c}$. However, their magnitudes reduced with an increase in $h/\bar{c}$, approaching the values of a normal hovering wing. The peak instantaneous lift and drag coefficients were observed at time *t*/*T* = 0.25. As expected, the cycle-averaged lift and drag coefficients decreased with $h/\bar{c}$. However, the SLE case experienced the highest mean lift coefficient at $h/\bar{c}=0.07$ and a lower mean lift coefficient when moved closer to the surface. On the other hand, the STE case experienced a monotonic decrease with $h/\bar{c}$, similar to that reported by the studies on the ground and ceiling effects. The peak at $h/\bar{c}=0.07$ observed in the SLE case might be attributed to the optimal space available to form a strong leading-edge vortex that is responsible for the high lift. Overall, the results indicate up to 60% enhancement in the aerodynamic forces when the wing moves closer to the water surface. This result will be useful for designers of underwater micro-air vehicles and flapping-wing experiments.

## Supporting information

S1 Dataset Directory containing all datasets. The compressed zip directory file contains all datasets presented in this article.
(ZIP)
